# The Promising Role of Selected Fibroblast Growth Factors as Potential Markers of Complications in Type 1 and Type 2 Diabetes

**DOI:** 10.3390/ijms26178754

**Published:** 2025-09-08

**Authors:** Elżbieta Cecerska-Heryć, Jaśmina Michałów, Weronika Engwert, Julia Marciniak, Radosław Birger, Natalia Serwin, Rafał Heryć, Aleksandra Polikowska, Małgorzata Goszka, Magda Wiśniewska, Barbara Dołęgowska

**Affiliations:** 1 Department of Laboratory Medicine, Pomeranian Medical University of Szczecin, Powstancow Wielkopolskich 72, 70-111 Szczecin, Poland; 83663@student.pum.edu.pl (J.M.); engwert.w@gmail.com (W.E.); juliamarciniak29@gmail.com (J.M.); radoslaw.birger@pum.edu.pl (R.B.); natalia.serwin@pum.edu.pl (N.S.); polikowska.aleksandra@gmail.com (A.P.); malgosia@goszka.pl (M.G.); barbara.dolegowska@pum.edu.pl (B.D.); 2 Cardiothoracic Surgery Clinic, Cardiac Rehabilitation Department, Pomeranian Medical University of Szczecin, Powstancow Wielkopolskich 72, 70-111 Szczecin, Poland; rafher87@wp.pl; 3 Department of Nephrology, Transplantology and Internal Medicine, Pomeranian Medical University of Szczecin, Powstancow Wielkopolskich 72, 70-111 Szczecin, Poland; magda.wisniewska@pum.edu.pl

**Keywords:** diabetes type 1, diabetes type 2, FGF-2, FGF-19, FGF-22, FGF-23, diabetic complications

## Abstract

Diabetes is a common chronic disease. Untreated diabetes may lead to complications such as nephropathy, neuropathy, retinopathy, and macroangiopathies. The main goal in treating diabetes is to limit the development of vascular complications. The FGF (fibroblast growth factor) family, with its potential as a biomarker for diabetic complications, offers a promising avenue for future research and treatment. The study aimed to analyze and compare the concentrations of selected fibroblast growth factors, FGF-2, FGF-19, FGF-22, and FGF-23, in the plasma of patients with type 1 and type 2 diabetes with those of the control group. The study group consisted of 73 patients, including 33 people with type 1 diabetes (18 M and 15 W) aged 18 to 68 years and 40 with type 2 diabetes (20 M and 20 W) aged 25 to 90. The control group consisted of 41 healthy individuals (23 men and 18 women) aged 21 to 56. The FGF-2, FGF-19, FGF-22, and FGF-23 concentrations were measured using ELISA. The study observed a significant relationship between the levels of FGF19 and FGF22 in the serum of patients with type 1 and type 2 diabetes, as well as in the control group (*p* < 0.001; *p* < 0.001). Statistical analysis revealed a significant relationship between FGF-2 and FGF-22 concentrations and hypertension (*p* = 0.03; *p* = 0.01). A statistically significant difference was also found between the concentrations of FGF-19 and FGF-22 (*p* = 0.001; *p* < 0.001) in the serum of people with normal weight and people with overweight and obesity. A significant correlation was also observed between the concentrations of FGF-22 and FGF-23 and arthritis (*p* = 0.01; *p* = 0.02). FGF-2, FGF-19, FGF-22, and FGF-23 likely significantly impact diabetes and its complications. In the future, they could serve as biomarkers for diabetic complications, aiding in diagnosis, patient monitoring, and even predicting potential complications for individuals. However, more research in this area is necessary.

## 1. Introduction

Diabetes is one of the most common chronic non-communicable diseases worldwide [1]. Its prevalence is steadily increasing, with 537 million adults affected globally in 2021 and projections estimating over 700 million cases by 2045 [2]. Diabetes results from both genetic and environmental factors and is characterized by chronic hyperglycemia due to insulin deficiency or resistance. Maintaining near-normal glucose levels remains the main therapeutic goal to prevent or delay complications [3].

According to the American Diabetes Association, diabetes is classified into four main categories: type 1, type 2, gestational, and other specific types [4]. Complications often accompany or follow the diagnosis and affect multiple organs, including the kidneys, cardiovascular system, retina, and nervous system [5]. These chronic micro- and macrovascular complications are multifactorial in origin and commonly linked to hyperglycemia, inflammation, oxidative stress, and dyslipidemia [6]. Their frequency varies depending on diabetes type and metabolic control, with nephropathy, retinopathy, macroangiopathy, and neuropathy being the most common [7]. Diabetes is the leading cause of chronic kidney disease, and patients are at significantly higher risk of cardiovascular events and mortality [1,2,8,9].

The fibroblast growth factor (FGF) family includes 23 polypeptides involved in diverse biological processes [7,10]. Based on structure and function, FGFs are divided into paracrine and endocrine subfamilies. Endocrine FGFs (e.g., FGF-19, FGF-21, FGF-23) regulate phosphate, lipid, and glucose metabolism, while paracrine FGFs (e.g., FGF-2) influence angiogenesis and tissue repair [10].

Several studies have shown that FGFs, particularly FGF-2, FGF-19, and FGF-23, are altered in diabetes and may be associated with complications such as nephropathy and impaired wound healing [8,11,12,13]. Endocrine FGFs (FGF-19, FGF-21) may also regulate glucose and lipid metabolism, acting as potential biomarkers in diabetes [8,14,15,16].

Based on literature data, FGF-2, FGF-19, FGF-22, and FGF-23 appear particularly relevant in the context of diabetic complications, especially those related to the kidneys and wounds. This study aimed to comprehensively evaluate the potential role of selected fibroblast growth factors—FGF-2, FGF-19, FGF-22, and FGF-23—as biomarkers of diabetic complications. Specifically, the objective was to analyze and compare the serum concentrations of these factors in individuals with type 1 and type 2 diabetes and in healthy controls. By assessing the relationships between FGF levels and selected clinical parameters, including BMI, age, comorbidities, and metabolic status, the study sought to determine whether these FGFs could serve as early indicators of diabetes-related complications, such as nephropathy, hypertension, joint degeneration, and dyslipidemia.

A further goal was to explore whether differences in FGF expression patterns may reflect underlying pathophysiological mechanisms specific to diabetes subtypes or disease severity. Identifying such associations could contribute to the development of improved diagnostic tools and individualized treatment strategies, with the long-term aim of enhancing clinical outcomes and preventing or mitigating the progression of diabetes complications.

## 2. Results

### 2.1. Characteristics of the Study Group

The study was conducted on a case–control basis. The study involved 73 patients with type 1 and type 2 diabetes, comprising 38 men and 35 women. These patients were split into two groups based on their type of diabetes. The first group consisted of 33 patients with type 1 diabetes (18 men and 15 women), aged 18 to 68 years (mean age, 40.1 ± 16.1 years). The second group consisted of 41 patients with type 2 diabetes (20 men and 21 women), aged 25 to 90 years (mean age, 71.8 ± 12.5 years). The control group consisted of 41 adults, including 23 healthy men and 18 healthy women, aged 21 to 56 (26.6 ± 8.23 years). The health status of the individuals in the study was confirmed by assessing basic biochemical parameters in the blood plasma, such as cholesterol, HDL, LDL, triglycerides, glucose, albumin, iron, uric acid, creatinine, and total protein, using reagent kits from BioMaxima S.A., Lublin, and the EnVision microplate reader (Perkin Elmer).

Inclusion criteria: study group—history of type 1 or type 2 diabetes; control group—healthy volunteers over 18 years of age, without chronic diseases such as diabetes, kidney, heart, liver disease, etc.

Exclusion criteria for the study included: 1. chronic diseases declared by the volunteers (e.g., diabetes, kidney disease, hepatic dysfunction); 2. infections; 3. operations performed within six months; 4. pregnancy and contraceptives; 5. severe coagulation disorders; 6. lack of written consent from the subjects to participate in the study. We obtained detailed information about the participants’ age, weight, height, waist circumference, disease duration, insulin and other medication use, diet, physical activity, combined therapy, diabetic complications, chronic diseases, recent hospitalizations, smoking status, hormonal contraception use, and other medications. The general characteristics of the study group are presented in Table 1, while the biochemical test results are detailed in Tables S1 and S2 (Supplementary Materials). Furthermore, detailed information regarding medications taken, the occurrence of diabetic complications, chronic diseases, and factors that could influence the test results is provided in Table 2, Table 3 and Table 4. Participants provided written informed consent and were informed about the study’s purpose and their right to withdraw at any time. The Pomeranian Medical University Bioethics Committee approved the study in Szczecin (Resolution No. KB 006/59/2022).

All procedures performed in this study were ethically governed by the institutional and/or national research committee, the 1964 Helsinki Declaration and its subsequent amendments, or comparable ethical standards.

### 2.2. Analysis of FGF Levels Comparison

**Figure 1:**
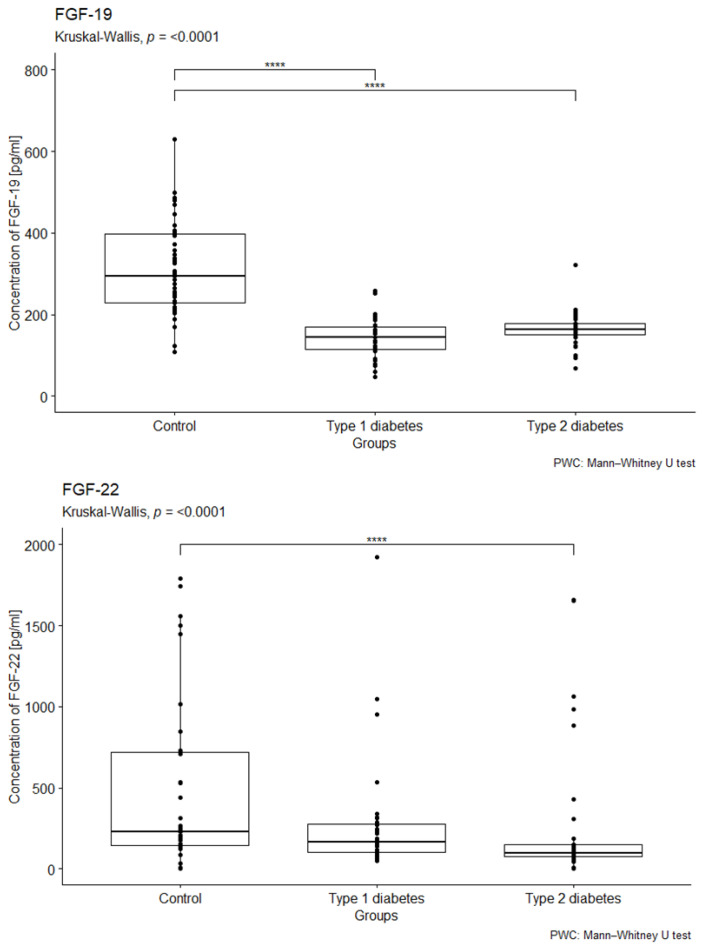
Comparison the concentrations of FGF-19 and FGF-22 (measured in pg/mL) in EDTA samples from individuals with type 1 diabetes, type 2 diabetes, and a control group. We conducted a Kruskal–Wallis rank ANOVA to assess the relationship between FGF-19 and FGF-22 concentrations and group membership, with results indicating statistical significance (*p* < 0.001). This was followed by post hoc analysis using the Bonferroni correction. The group composition included 41 individuals in the control group, 33 individuals with type 1 diabetes, and 40 individuals with type 2 diabetes. **** *p* < 0.0001.

The study observed a difference in the relationship between the median serum concentrations of FGF19 and FGF22 in patients with type 1 and type 2 diabetes, as well as in the control group (*p* < 0.001; *p* < 0.001). However, there were no significant differences in the concentrations of FGF-2 between the study and control groups (see Figure S1, Supplementary Materials). Furthermore, the concentration of FGF-19 was significantly lower in both study groups compared to the control group, with the lowest concentration found in patients with type 1 diabetes (see Figure 1). The post hoc analysis also revealed a notable difference in the FGF-19 concentration between the control group and patients with type 1 diabetes (*p* < 0.001). Additionally, the study group with type 2 diabetes showed a significantly lower FGF-22 concentration compared to the control group (*p* < 0.001) (see Figure 1). Lastly, there were no statistically significant differences in FGF-23 concentrations between the study and control groups (see Figure S1 in the Supplementary Materials). For detailed results of the fibroblast growth factor concentrations, including their mean, standard deviation, median, lower and upper quartiles, and IQR, please refer to Table S3 (Supplementary Materials).

#### 2.2.1. Analysis of the Relationship Between FGF Concentration in the Serum of Diabetic Patients with Hypertension

**Figure 2:**
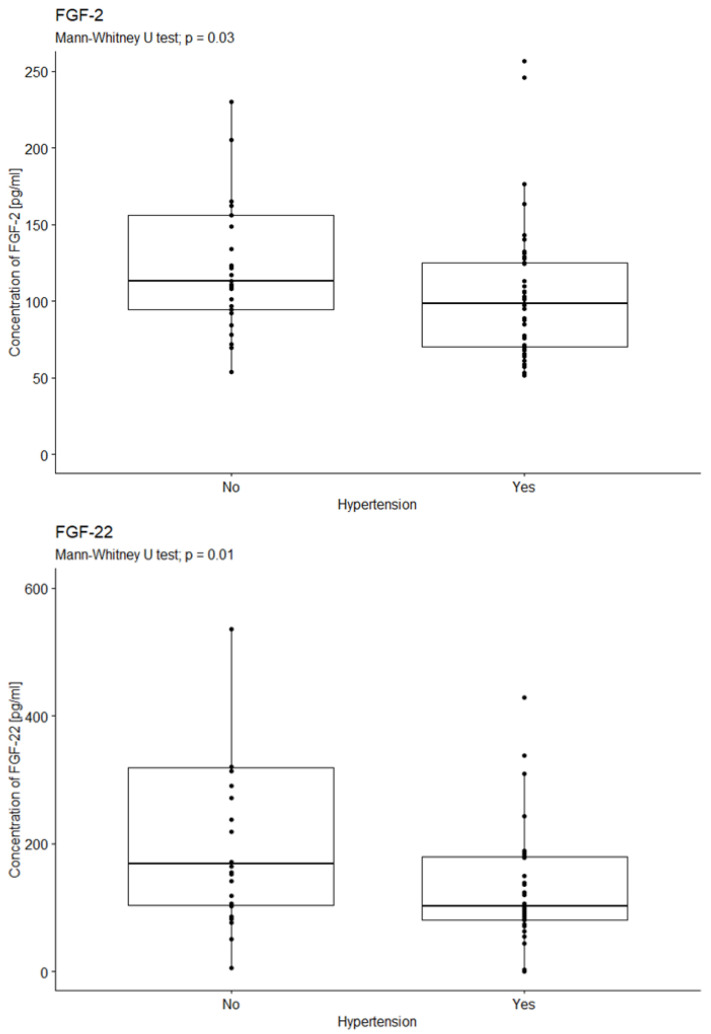
Comparison of FGF-2 and FGF22 concentration according to the occurrence of hypertension. U-test analysis of the concentration of FGF-2 in patients with and without hypertension, *p* = 0.03; *p* = 0.01. Yes—47 patients; No—26 patients.

The influence of hypertension on the median concentrations of the tested factors was examined (hypertension—yes/no). The statistical analysis revealed statistically significant differences in median FGF-2 concentrations between patients with and without hypertension (*p* = 0.03), with lower median levels observed in patients with hypertension (see Figure 2). Similarly, statistically significant differences in median FGF-22 concentrations were found in relation to hypertension status (*p* = 0.01). Patients with hypertension exhibited lower median levels of FGF-22 compared to individuals without hypertension (see Figure 2).

#### 2.2.2. Analysis of the Relationship Between FGF Concentration in the Serum of Diabetic Patients and BMI

The study examined the impact of BMI on the median concentrations of the investigated factors. The normal BMI range was defined as 18.5–24.9, overweight as 25–29.9, and obesity as a BMI of 30 or higher.

The statistical analysis did not reveal statistically significant differences in median FGF-2 concentrations across BMI categories (*p* = 0.93; see Figure S2 in the Supplementary Materials).

**Figure 3:**
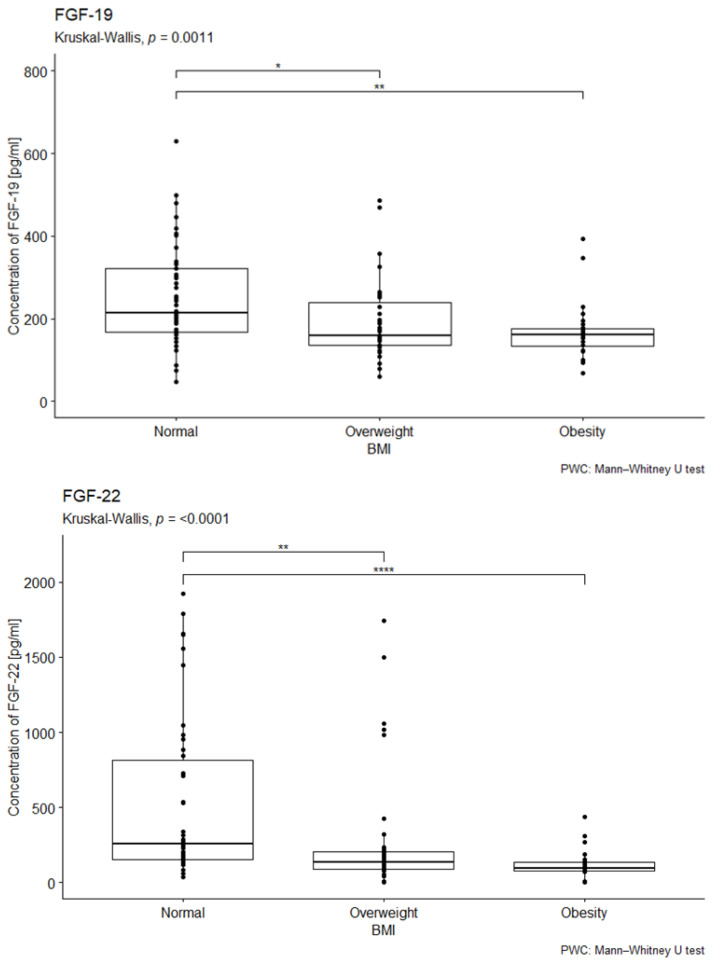
Comparison of FGF-19 and FGF-22 concentration by body mass index. U-test analysis of the concentration of FGF-19 in people with normal, overweight, and obese BMI, *p* = 0.001, *p* < 0.001, followed by post hoc analysis with the Bonferroni correction. Normal—53 people; Overweight—35 people; Obesity—28 people. * p < 0.05, ** *p* < 0.01, **** *p* < 0.0001.

In contrast, statistically significant differences in median FGF-19 concentrations were observed among individuals with different BMI values (*p* = 0.038 in the overall cohort; *p* = 0.001 in patients with diabetes). Overweight and obese patients exhibited lower median FGF-19 concentrations compared to the control group (see Figure 3). Post hoc analysis with Bonferroni correction further demonstrated significantly lower FGF-19 concentrations in diabetic patients with overweight (*p* < 0.01) and obesity (*p* < 0.001) compared to those with a normal BMI (see Figure 3).

A statistically significant difference in median FGF-22 concentrations was also found depending on BMI status (*p* < 0.001). Lower median FGF-22 concentrations were observed in both overweight and obese patients compared to the control group (*p* < 0.001 for both; see Figure 3).

No statistically significant differences in median FGF-23 concentrations were detected across BMI categories in diabetic patients (*p* = 0.3). Similarly, no significant differences were observed between overweight or obese patients and the control group (see Figure S2 in the Supplementary Materials).

### 2.3. Analysis of the Relationship Between FGF Concentration in the Serum of Diabetic Patients with Joint Degeneration

**Figure 4:**
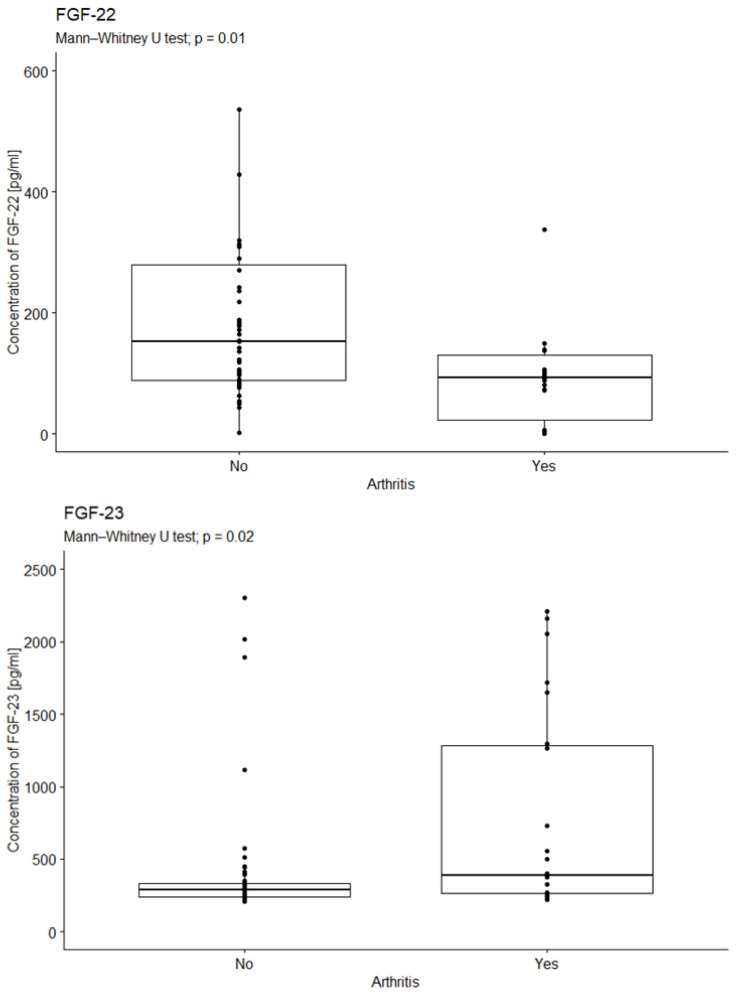
Comparison of FGF-22 concentrations due to arthritis. U-test analysis of the concentration of FGF-22 in patients with and without arthritis, *p* = 0.01; *p* = 0.02. Yes—15 patients; No—58 patients.

The study investigated the impact of diabetes-related joint degeneration on specific bodily factors. The analysis revealed a significant difference in median FGF-22 concentrations between FGF-22 concentration and arthritis (*p* = 0.01). Patients with joint degeneration exhibited lower FGF-22 concentrations compared to those without (Figure 4).

**Figure 5:**
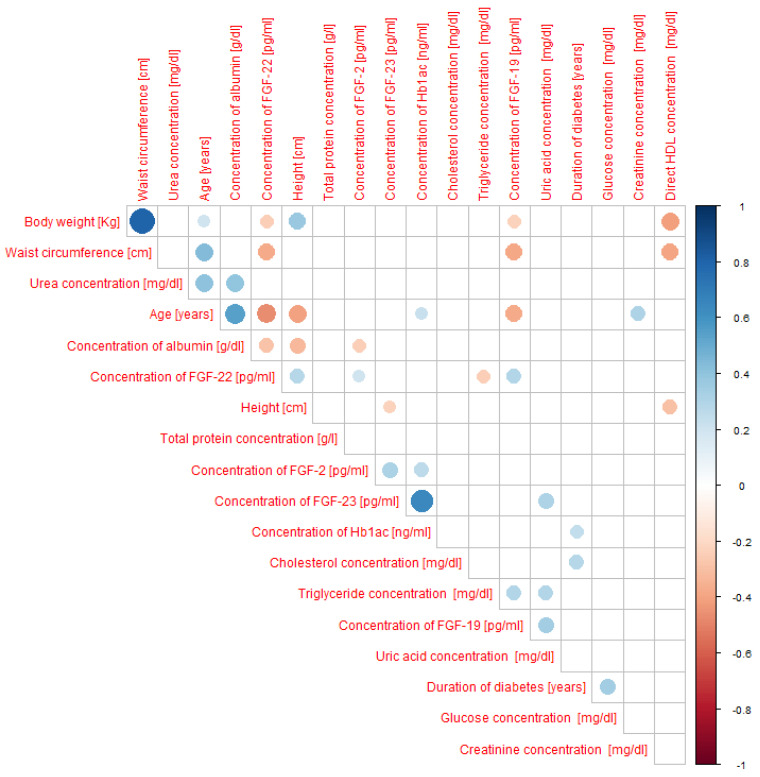
Spearman’s rank correlation between the concentrations of selected fibroblast growth factors (FGF-2, FGF-19, FGF-22, FGF-23), anthropometric parameters, and biochemical indicators in all study participants (including patients with type 1 and type 2 diabetes as well as controls). N-114 people.

The statistical analysis revealed a significant difference in median FGF23 concentration between those with and without joint degeneration (*p* = 0.0213). Patients with joint degeneration exhibited higher levels of FGF-23 than those without (see Figure 5).

### 2.4. Spearman’s Rank Correlation Analysis

We observed significant correlations, as indicated by Spearman’s rank correlation coefficient, denoted as “r.” Regarding FGF-2 concentration, we found a weak, positive correlation with glycated hemoglobin (r = 0.27) and a weak, negative correlation with albumin concentration (r = −0.24). For FGF-19 concentration, we found a weak, negative correlation with body weight (r = −0.23) and waist circumference (r = −0.39) and a weak, positive correlation with uric acid concentration (r = 0.34) and triglyceride concentration (r = 0.30). As for FGF-22 concentration, we observed a moderate negative correlation with age (r = −0.47), and weak, negative correlations with body weight (r = −0.25), waist circumference (r = −0.37), albumin concentration (r = −0.28), and triglyceride concentration (r = -0.24), and a weak, positive correlation with growth (r = 0.29). Finally, in the case of FGF-23 concentration, we found a moderate, positive correlation with glycated hemoglobin concentration (r = 0.64) and a weak, positive correlation with uric acid concentration (r = 0.30). All correlations are displayed in Figure 5.

Due to a statistically significant difference in age between study groups, Spearman’s rank correlation analysis was performed to assess the potential relationship between age and the concentrations of selected fibroblast growth factors (FGF-2, FGF-19, FGF-22, and FGF-23).

Spearman’s rank correlation analysis revealed several significant associations between fibroblast growth factors (FGFs) and clinical parameters.

In the entire study cohort (n = 114), age was negatively correlated with FGF-19 (ρ = −0.28, *p* = 0.004) and FGF-22 (ρ = −0.45, *p* < 0.001), while no significant associations were observed with FGF-2 or FGF-23. BMI was also inversely correlated with FGF-19 (ρ = −0.28, *p* = 0.005) and FGF-22 (ρ = −0.46, *p* < 0.001).

When analyzed separately by diabetes type, in patients with type 2 diabetes (n = 73), age showed negative correlations with FGF-2 (ρ = −0.27, *p* = 0.022) and FGF-22 (ρ = −0.32, *p* = 0.005). BMI was also negatively correlated with FGF-22 (ρ = −0.42, *p* < 0.001).

In patients with type 1 diabetes (n = 31), age correlated negatively with FGF-22 (ρ = −0.39, *p* = 0.027). Additionally, a positive correlation was found between BMI and FGF-19 (ρ = 0.35, *p* = 0.05).

In the control group (n = 41), BMI correlated negatively with FGF-19 (ρ = −0.35, *p* = 0.022), while no significant associations were observed for age with any of the FGFs.

Overall, these results indicate that both age and BMI are consistently associated with reduced concentrations of FGF-19 and FGF-22, particularly in patients with type 2 diabetes, whereas FGF-2 and FGF-23 showed no consistent correlations with either parameter.

All correlations are displayed in Table 5.

Given the clinical relevance of glycemic control, Spearman’s rank correlation analysis was conducted to assess the relationship between glycated hemoglobin (HbA1c) levels and concentrations of selected fibroblast growth factors (FGF-2, FGF-19, FGF-22, and FGF-23) in patients with diabetes (*n* = 73).

A statistically significant and strong positive correlation was observed between HbA1c and FGF-23 levels (*r* = 0.64, *p* < 0.001), suggesting that FGF-23 concentrations increase with worsening glycemic control. This may indicate a potential role for FGF-23 as a marker of metabolic dysregulation in diabetes.

No significant correlations were found between HbA1c and FGF-2 (*r* = 0.16, *p* > 0.05), FGF-19 (*r* = −0.09, *p* > 0.05), or FGF-22 (*r* = −0.13, *p* > 0.05), although the weak trends observed may warrant further investigation in larger cohorts. All correlations are displayed in Table 6.

These findings emphasize the specific association between FGF-23 and long-term glucose homeostasis, highlighting its potential value in monitoring or stratifying diabetes-related complications.

### 2.5. ANCOVA and a Multivariate Regression Analysis

In our analysis, we conducted a multivariate regression to investigate how diabetic complications/comorbidities (independent variables) affect the concentration of fibroblast growth factors FGF-2, FGF-19, FGF-22, and FGF-23 (dependent variables). We found that urinary tract diseases significantly impacted FGF-2, explaining 26% of the variation and increasing its concentration by 0.56 pg/mL (Table 7). Additionally, gout affected the concentration of FGF-23 by approximately 30%, resulting in a decrease of 0.35 pg/mL (Table 8).

ANCOVA (analysis of covariance) was conducted to adjust for the effects of covariates (age, BMI) and comorbidities on the levels of the studied FGF factors. The results of this analysis are shown in Table 9. An ANCOVA analysis of covariance was performed to control for the influence of covariates (age and BMI) and comorbidities on the levels of the studied FGF factors. The ANCOVA analysis revealed that group membership had a statistically significant effect on FGF-19 levels (F = 3.362, *p* = 0.038). In contrast, neither age (F = 0.842, *p* = NS), BMI (F = 0.708, *p* = NS), nor comorbidities such as urinary tract diseases (F = 2.440, *p* = NS) and joint degeneration (F = 0.034, *p* = NS) had a significant influence on FGF-19. These findings indicate that differences in FGF-19 concentrations are primarily associated with diabetes type or health status, rather than demographic factors or the presence of comorbidities.

In contrast, FGF-23 levels were strongly influenced by comorbidities, particularly urinary tract diseases (*F* = 72.330, *p* < 0.001) and joint degeneration (*F* = 550.575, *p* < 0.001). However, age (*F* = 0.118, *p* = NS) and BMI (*F* = 2.604, *p* = NS) did not significantly impact FGF-23 concentrations. These results suggest that elevated FGF-23 levels are more closely linked to specific pathological conditions rather than general anthropometric characteristics or group classification.

Overall, the analysis confirmed that age and BMI were not confounding variables for either FGF-19 or FGF-23, supporting the robustness of the observed associations.

The results of the analysis are presented in Table 6.

## 3. Discussion

Diabetes is one of the most widespread chronic diseases worldwide, with projections indicating over 700 million cases by 2045, representing a 50% increase from 2017 [2]. Severe complications, including retinopathy, neuropathy, nephropathy, and diabetic foot, remain leading causes of morbidity and mortality in these patients [17]. The mechanisms of hyperglycemia-induced vascular damage are complex and not fully understood, but elevated intracellular glucose is believed to increase reactive oxygen species and disrupt critical signaling pathways [18]. Diabetes represents a spectrum of metabolic disorders with hyperglycemia as the unifying feature, and vascular injury is further aggravated by comorbidities such as hypertension and obesity. Identifying prognostic biomarkers is therefore essential for predicting complications and enabling early intervention. Such markers may improve risk stratification, guide treatment, and enhance patient outcomes [17].

Fibroblast growth factors (FGFs) have emerged as promising candidates in this context. They play key roles in metabolic homeostasis and cellular processes, and altered expression has been linked to diabetes, its complications, and comorbid obesity. Preclinical studies have highlighted the diagnostic and therapeutic potential of these compounds, with ongoing clinical trials further exploring their role. Reports suggest that FGFs modulate insulin resistance induced by high-fat diets, fasting glucose, and triglyceride levels, with FGF-21 being the most extensively studied member of this family [2,19].

In our study, the levels of FGF-2, FGF-19, FGF-22, and FGF-23 were measured in patients with type 1 and type 2 diabetes and compared with healthy controls. We found a significant association between FGF-2 concentration and hypertension, with lower levels observed in hypertensive patients. Cho et al. [20] linked the FGFRL1 gene, which interacts with FGF-2, to hypertension, identifying two relevant polymorphisms (rs13143527 and rs55639339). Dono et al. [21] demonstrated that FGF-2 knockout mice exhibited reduced vascular tone and arterial pressure, while our study paradoxically found increased blood pressure in FGF-2–deficient individuals.

Altered glucose metabolism also appears to affect FGF-2 activity. Facchiano et al. [22] demonstrated that rapid glucose turnover in diabetic mice resulted in glycosylated FGF-2, which impaired receptor binding and signaling. Similarly, Huang et al. [23] found that reduced FGF-2 expression was associated with impaired fracture healing in type 2 diabetes, a finding confirmed in osteoblast cultures under hyperglycemic conditions. FGF-2 may also be important in wound repair, as demonstrated by Hiller et al. [24], who showed its role in granulation, vascularization, and epithelial regeneration in diabetic foot ulcers; however, our cohort was too small (n = 5) to confirm this.

We also observed a weak positive correlation between FGF-2 and HbA1c, the gold standard for long-term glycemic control, which is strongly linked to the risk of complications [25]. Moreover, our finding of reduced FGF-2 in patients with both diabetes and hypertension is notable. While FGF-2 overproduction has been reported in pulmonary hypertension [26], its role in neurogenic blood pressure regulation [27] and hypertension-induced renal injury [28] is well established. However, our data suggest an opposite trend. This discrepancy may reflect methodological differences or indicate a slower progression of hypertension in our cohort.

In this study, FGF-19 concentrations were significantly lower in both type 1 and type 2 diabetes compared to the control group, consistent with previous reports linking reduced FGF-19 levels to diabetes [29,30]. Experimental studies further suggest a metabolic role for FGF-19. In obesity models, it reduced body weight, blood glucose, and insulin levels [31]. In contrast, FGF-19 transgenic mice exhibited increased metabolic rate and resistance to diet-induced obesity [32]. Mechanistically, FGF-19 crosses the blood–brain barrier to stimulate energy expenditure and reduce glycemia, and in peripheral tissues it enhances insulin sensitivity and glucose uptake. In the liver, FGF-19 is induced by postprandial bile acids, suppresses bile acid synthesis, and promotes the synthesis of glycogen and protein [33].

Clinical observations support these findings. Wang et al. [34] demonstrated that women with gestational diabetes had lower FGF-19 levels and higher FGF-21 levels, while Mraz et al. [35] found an association between reduced FGF-19 and acute hyperglycemia. In line with these reports, our cohort showed low FGF-19 levels in both diabetes groups (average glucose: 174 mg/dL in T1D, 142 mg/dL in T2D). Lundasen et al. [36] demonstrated that FGF-19 fluctuates with feeding, peaking postprandially, while fasting levels remain relatively stable [37].

We also found that overweight and obese patients had reduced FGF-19 compared to controls, in agreement with Gómez-Ambrosi et al. [38]. Moreover, although FGF-19 does not naturally occur in mice, Tomlinson et al. [32] demonstrated that its activation lowered plasma levels of glucose, triglycerides, and cholesterol. In our study, a weak positive correlation was observed between FGF-19 and triglycerides, which contrasts with the weak negative correlation reported by Stejskal et al. [39]. Finally, we observed a weak positive correlation between FGF-19 and uric acid, a novel finding, though a similar association has been reported for FGF-23 [40].

In our study, FGF-22 concentrations differed significantly between groups, with lower levels observed in individuals with type 2 diabetes compared to those in the control group. To date, there are no published reports on FGF-22 levels in diabetic patients, and its physiological role remains under investigation [19].

Although the molecular mechanisms of FGF-22 are not fully elucidated, its involvement in depression has been reported. In a rat model of chronic mild stress, FGF-22 levels correlated negatively with interleukin-1β (IL-1β); exogenous FGF-22 administration alleviated depressive symptoms, likely via suppression of IL-1β expression [41]. This is noteworthy, as interleukins are central markers of inflammation, also present in diabetes, and represent key targets of pharmacological and insulin-based therapies. Indeed, inhibition of pro-inflammatory cytokines has been confirmed in experimental models [42], and a meta-analysis by Jin et al. [43] demonstrated that elevated IL-6 and IL-1β have predictive value in type 1, type 2, and gestational diabetes.

Given the limited number of studies on FGF-22 in the context of diabetes, its role remains largely unexplored. However, based on emerging evidence from experimental models, it is reasonable to hypothesize that FGF-22 may interact with inflammatory mediators involved in the pathogenesis of diabetes-related complications. One possible mechanism is the interplay between FGF-22 and pro-inflammatory cytokines, such as interleukin-1β (IL-1β), which are known to contribute to insulin resistance, beta-cell dysfunction, and vascular inflammation in diabetes [44,45]. These associations suggest that FGF-22 may exert anti-inflammatory effects or play a role in immunometabolic regulation.

The observed association between FGF-22 and interleukin-1β in depression suggests a potential role in diabetes-related inflammation. In our study, patients with diabetes and coexisting mood disorders exhibited higher levels of FGF-2 and FGF-23. However, these findings were not statistically significant, likely due to the small sample size and possible underreporting of depressive symptoms. Nevertheless, this warrants further investigation.

We also found that patients with joint degeneration had significantly lower FGF-22 concentrations compared to those without, a novel observation not previously described in the literature but worth exploring in future studies.

Moreover, FGF-22 levels were significantly lower in overweight and obese individuals compared to controls, with a clear trend of decreasing concentration as BMI increased. Levels were also reduced in type 2 diabetes, which may reflect the contribution of obesity to this condition. Since FGFs are closely linked with inflammation associated with hyperglycemia [46,47], it is plausible that adipose tissue inflammation plays a key role. Obesity induces phenotypic changes in white adipose tissue, characterized by dysfunctional adipocytes that secrete pro-inflammatory cytokines, impairing both local adipose tissue function and distant organ systems [48].

In this context, the inverse relationship observed between FGF-22 and BMI may reflect an underlying mechanism in which FGF-22 is downregulated in states of chronic low-grade inflammation, such as obesity. This supports the need for further mechanistic studies to clarify whether FGF-22 plays a compensatory, regulatory, or pathogenic role in the immunometabolic axis.

In the case of FGF-22, a weak, positive correlation with body height was observed. The result is consistent with a previous study by Cho et al. [20], which found an association between the FGFRL1 gene and osteoporosis, hypertension, and height, as well as an interaction between FGFRL1 and FGF genes, including the FGF-22 gene. The results suggest that the FGFRL1 gene and associated genes may determine height. A single-nucleotide polymorphism in the FGF-22 gene, associated with hypertension and height, is rs8109113. In the case of FGF-22 concentration, a moderate negative correlation was observed with age, and a weak negative correlation was observed with both albumin and triglyceride concentrations, which is not reported in the literature.

Similar to FGF-2, lower FGF-22 levels were observed in patients with diabetes and hypertension; however, no prior reports have linked FGF-22 to hypertension. By contrast, several studies have described associations between FGF-23 and vascular complications. Miri et al. demonstrated elevated FGF-23 levels in patients with end-stage renal disease (ESRD) and pulmonary hypertension, suggesting a role for FGF-23 in vascular pathology [49]. In contrast, Kendrick et al. found an association between higher plasma FGF-23 levels and mortality, cardiovascular events, and the initiation of dialysis [50]. Our findings, however, did not replicate these results, which may reflect slower disease progression in our cohort.

In our study, no significant differences in FGF-23 levels were observed between diabetic patients and controls. Nonetheless, FGF-23 is considered a biomarker of cardiovascular risk, chronic kidney disease, and mortality, with evidence linking it to diabetic nephropathy progression [11]. Associations with uric acid secretion have also been reported in type 2 diabetes [49], and elevated FGF-23 is consistently related to poor outcomes [50].

Rodelo-Haad et al. [51] showed that very high FGF-23 levels characterize chronic kidney disease and may drive inflammation, impaired neutrophil function, infections, left ventricular hypertrophy, myocardial infarction, and heart failure. Similarly, Yeung et al. [17] reported that FGF-23 is elevated in type 2 diabetes independently of renal function and increases cardiovascular risk, although the effect is smaller than in advanced CKD. Wahl et al. [52] further confirmed that diabetes predicts higher FGF-23 levels, faster renal decline, and higher risks of cardiovascular disease and mortality.

Although we did not observe these associations, possibly due to limited sample size, disease duration, or age distribution, we found a moderate positive correlation between FGF-23 and HbA1c, and a weak positive correlation with uric acid, consistent with earlier findings by Asicioglu et al. [40].

In our study, patients with joint degeneration had significantly higher FGF-23 levels, consistent with the findings of Orfanidou et al. [53], who reported increased FGF-23 expression in osteoarthritic chondrocytes compared to healthy ones. Wang et al. [54] demonstrated that BMI and HbA1c independently influence FGF-1 in type 2 diabetes. We observed similar associations: HbA1c with FGF-2 and FGF-23, BMI with FGF-19 and FGF-22, and uric acid with both FGF-19 and FGF-23.

FGF-21, known to regulate glucose and lipid metabolism, was previously reported to correlate positively with triglycerides in type 2 diabetes [55]; a similar relationship with triglycerides was observed in our study for FGF-19 and FGF-22. Elevated FGF-21 levels have been recognized as a potential therapeutic target in metabolic disease, particularly in individuals with impaired glucose tolerance and diabetes [56].

We also found that urinary tract diseases significantly increased FGF-2 concentrations by 26% (0.56 pg/mL). This finding is consistent with that of Livingston et al. [54], who demonstrated that renal tubular cells produce FGF-2, thereby driving fibroblast activation and interstitial fibrosis during maladaptive renal repair. Finally, we identified a novel association between gout and reduced FGF-23 levels (–30%, approximately –0.35 pg/mL), an observation that warrants further investigation.

Importantly, the clinical applicability of fibroblast growth factors (FGFs) as potential prognostic biomarkers deserves further attention. The observed alterations in FGF-2, FGF-19, FGF-22, and FGF-23 concentrations—particularly their associations with hypertension, obesity, nephropathy, and joint degeneration—support their role not only in the pathogenesis but also in the clinical stratification of diabetic complications. The integration of FGF measurements into routine diagnostic panels, alongside established markers such as HbA1c and eGFR, may enhance early risk assessment and enable more personalized therapeutic decisions [20,57]. For instance, the identification of reduced FGF-19 or FGF-22 levels in overweight individuals or those with type 2 diabetes could indicate a higher risk of metabolic decompensation and musculoskeletal deterioration [58,59]. Similarly, elevated FGF-23 levels in patients with joint degeneration or urinary tract comorbidities may flag early organ involvement or subclinical renal impairment, as suggested by recent findings in both renal transplant recipients and otherwise healthy individuals [53,60]. Incorporating such biomarkers into clinical practice could therefore help clinicians move beyond glycemic control alone and adopt a more comprehensive, complication-oriented management approach for patients with diabetes [50,61,62].

This study has several significant limitations that should be taken into account when interpreting the findings. First and foremost, the relatively small sample size restricts the statistical power and generalizability of the results. Additionally, the diversity within the study population complicates interpretation, as many participants experienced severe comorbidities unrelated to diabetes, including neurological, endocrine, immunological, and respiratory conditions. These individuals were also undergoing various pharmacological treatments (e.g., levetiracetam, lamotrigine, budesonide, formoterol, levothyroxine, methotrexate), which could potentially influence inflammatory or metabolic parameters. While we excluded patients with coagulation disorders or those on chronic anticoagulant therapy, the overall range of medication use remains a potential confounding factor that may have impacted FGF concentrations. Another significant limitation is the difference in age and Body Mass Index (BMI) between the groups. It is inherent in comparing populations with type 1 and type 2 diabetes that patients with type 2 diabetes are often older and have higher BMI. Although we attempted to address this issue by including a broad age range in the control group and by applying ANCOVA to control covariates such as age and BMI, we cannot entirely rule out residual confounding. Therefore, the correlations between FGF-19, FGF-22, and age or BMI must be interpreted cautiously. Moreover, the inclusion of patients with advanced complications or those on multiple drug regimens introduces additional variability. While these real-world conditions reflect the complexity of diabetes management, they also limit our ability to isolate diabetes-specific mechanisms that affect FGF levels. Consequently, although the findings provide valuable insights, their applicability to broader, treatment-naive, or early-stage diabetic populations may be limited. To improve future analyses, it would be beneficial to conduct sensitivity analyses stratified by comorbidity burden or medication use. Such analyses would help clarify the robustness of the observed associations and evaluate the extent to which these variables influence FGF levels. This approach may enable us to determine whether diabetes-related factors or broader systemic influences primarily drive the detected differences. In light of these limitations, further large-scale studies with more homogeneous cohorts, incorporating medication tracking, and longitudinal follow-up are necessary to validate the current results and clarify the mechanistic and prognostic roles of FGF-2, FGF-19, FGF-22, and FGF-23 in diabetes.

## 4. Materials and Methods

### 4.1. Material

The blood sample was taken from a vein in the arm and placed in tubes containing K_2_EDTA to prevent clotting. After collection, the blood was centrifuged for 10 min at 2600 rpm and 20 °C. The resulting plasma and serum were transferred to new tubes and stored at −80 °C until analysis. The analysis included testing for glucose, cholesterol, triglycerides, direct HDL, uric acid, urea, albumin, total protein, creatinine, and growth factors.

### 4.2. Determination of FGF-2 Concentration

The concentration of FGF-2 was determined using the enzyme-linked immunosorbent assay (ELISA) method, with a ready-made Human FGF-2 ELISA Kit from ELK Biotechnology (Wuhan, China). 0.1 mL of plasma was added to wells coated with FGF-2-specific antibodies and standard solutions. The plates were incubated for 80 min at 37 °C. In the next step, the wells were washed three times with the washing solution, 0.2 mL each. Then 0.1 mL of a biotin-conjugated antibody solution was added to the wells and incubated for 50 min at 37 °C. After incubation, the wells were washed 3 times with 0.2 mL each. Then 0.1 mL of streptavidin conjugate and HRP (horseradish peroxidase) was added and incubated for 50 min at 37 °C. After incubation, the sample was washed five times with washing buffer. Then, 0.2 mL and 0.09 mL of TMB (tetramethylbenzidine) solution were added and incubated in the dark for 20 min at 37 °C. After incubation, 0.05 mL of stop solution was added to terminate the enzyme-substrate reaction. Absorbance readings were taken at a wavelength of 450 nm using an EnVision microplate reader (Perkin Elmer, Springfield, IL, USA). The FGF-2 concentration was calculated based on a standard curve prepared using the concentrations of standard samples.

### 4.3. Determination of FGF-19 Concentration

The concentration of FGF-19 was determined using the ELISA method with the ready-made Human FGF-19 ELISA Kit from ELK Biotechnology (Wuhan, China). 0.1 mL of plasma and standard solutions were added to the wells coated with FGF-19-specific antibodies. The plates were incubated for 80 min at 37 °C. In the next step, the wells were washed three times with the washing solution, 0.2 mL each. Then 0.1 mL of a biotin-conjugated antibody solution was added to the wells and incubated for 50 min at 37 °C. After incubation, the wells were washed 3 times with 0.2 mL each. Then, 0.1 mL of HRP-conjugated streptavidin conjugate was added and incubated for 50 min at 37 °C. After incubation, the washing buffer was repeated, this time 5 times, with 0.2 mL each. Then, 0.09 mL of TMB solution was added, and the mixture was incubated in the dark for 20 min at 37 °C. After incubation, 0.05 mL of stop solution was added to terminate the enzyme-substrate reaction. Absorbance readings were taken at a wavelength of 450 nm using an EnVision microplate reader (Perkin Elmer) Springfield, IL, USA). FGF-19 concentration was calculated based on a standard curve prepared using the concentrations of standard samples.

### 4.4. Determination of FGF-22 Concentration

The concentration of FGF-22 was determined using the ELISA method with the Human FGF-22 ELISA Kit from Reed Biotech Ltd. (Wuhan, China). 0.1 mL of plasma and standard solutions were added to wells coated with FGF-22-specific antibodies. The plates were then incubated for 90 min at 37 °C. After incubation, the wells were washed three times with 0.3 mL of washing solution each time. Next, 0.1 mL of a biotin-conjugated antibody solution was added to the wells and incubated for 60 min at 37 °C. Following another round of washing, 0.1 mL of HRP conjugate was added and incubated for 30 min at 37 °C, followed by another round of washing. Then, 0.1 mL of substrate was added and incubated for 15 min at 37 °C. After this, 0.05 mL of stop solution was added to halt the reaction. The absorbance readings were taken at a wavelength of 450 nm using an EnVision microplate reader (Perkin Elmer), Springfield, IL, USA). Finally, the concentration of FGF-22 was calculated based on a standard curve prepared using the concentrations of standard samples.

### 4.5. Determination of FGF-23 Concentration

The concentration of FGF-23 was determined using the ELISA method with the Human Fibroblast Growth Factor-23 ELISA Kit from BT LAB (Wuhan, China). To conduct the test, 0.04 mL of plasma was mixed with 0.01 mL of anti-FGF-23 antibody and 0.05 mL of standard solutions in wells coated with FGF-23-specific antibodies. Then, 0.05 mL of HRP-conjugated streptavidin conjugate was added and incubated for 60 min at 37 °C. After incubation, the wells were washed five times with 0.3 mL of washing buffer. Subsequently, 0.05 mL of substrate A and 0.05 mL of substrate B were added and incubated in the dark for 10 min at 37 °C. Then, 0.05 mL of stop solution was added to halt the enzyme-substrate reaction. Absorbance readings were taken at a wavelength of 450 nm using an EnVision microplate reader from Perkin Elmer, Springfield, IL, USA. The concentration of FGF-23 was calculated based on a standard curve prepared using concentrations of standard samples.

### 4.6. Statistical Analysis

The results of the measurements were statistically analyzed using RStudio (Version 2023.6.0.421) and Statistica 13 PL Trial. Quantitative results were presented as mean ± standard deviation. The Shapiro–Wilk test was used to assess normality, along with the calculation of skewness and kurtosis values. Skewness and kurtosis values ranging from −2 to +2 indicated a normal distribution. The Shapiro–Wilk test results revealed that most variables exhibit distributions that deviate from normal, but not the kurtosis and skewness values. Each parameter tested was described using statistical measures, including sample size, arithmetic mean, median, standard deviation, and minimum and maximum values. Levene’s test was applied to determine the equality of variances, which indicated homoscedasticity. Fisher’s exact test and Chi-square test were used to analyze qualitative data such as smoking status, sex, and treatment strategy. Since all data distributions are non-parametric, the Kruskal–Wallis test was used for unrelated variables. To confirm the significance of the results, the U-Mann–Whitney post hoc test was used. Post hoc analyses were performed with Bonferroni correction to adjust for multiple comparisons. A statistically significant result indicates a difference between the groups. Spearman’s rank correlation coefficient was used to measure the strength of correlation, especially for data that do not have a normal distribution. Furthermore, multivariate regression and ANCOVA analysis were performed to investigate how diabetic complications/comorbidities (independent variables) affect the concentration of fibroblast growth factors FGF-2, FGF-19, FGF-22, and FGF-23 (dependent variables).

The G*Power software version 3.1.9.2 performed a sensitivity analysis for Kruskal–Wallis ANOVA. Based on our analysis, a Kruskal–Wallis ANOVA with 114 participants across 3 groups would be sensitive to the effects of η^2^ = 0.113 with a power of 95% (*p* = 0.05). This indicates that our study would not be equipped to detect an impact smaller than η2 = 0.113 reliably. For all analyses, an alpha level of 0.05 was applied. A *p*-value of less than 0.05 indicated a statistically significant result.

## 5. Conclusions

The findings of this study suggest that fibroblast growth factors, specifically FGF-19 and FGF-22, may play crucial roles in the pathophysiology of both type 1 and type 2 diabetes. In diabetic patients, the observed lower concentrations of FGF-2 and FGF-22 could indicate a significant association with hypertension, emphasizing the interconnected nature of these metabolic conditions. Furthermore, the study highlights that reduced levels of FGF-19 and FGF-22 in individuals suffering from obesity may reflect a more profound link between these growth factors and excess body weight.

Additionally, the analysis reveals that decreased levels of FGF-22 and FGF-23 in diabetic patients with joint degeneration may serve as potential biomarkers for this specific complication. These findings underscore the need for further research to explore the intricate roles of fibroblast growth factors, which could ultimately provide valuable insights into their utility as biomarkers for the complications associated with diabetes.

## Figures and Tables

**Table 1:** General characteristics of people qualified for the study.

Parameter	Control	Diabetes Type 1	Diabetes Type 2	*p* *	*p* **
Sex	F-18 M-23	F-15 M-18	F-21 M-20	NS	NS
Age [years]	26.6 ± 8.23	40.1 ± 16.1	71.8 ± 12.5	<0.0001	<0.0001
BMI	23.7 ± 3.82	25.3 ± 4.85	31.9 ± 7.18	<0.0001	<0.0001
Duration of the disease	-	19.5 ± 14.8	13.4 ± 10.7	NS	-

*p*—*p*-value*; p* */*p* **—statistical significance for differences between the Control group and Diabetes type1/type2 groups; for quantitative variables—Kruskal–Wallis rank ANOVA; and Chi-square/Fisher’s exact test for qualitative variables. NS—Not Significant.

**Table 2:** Characteristics of the study group in terms of medication use.

Medications Taken	Diabetes Type 1			Diabetes Type 2		
	Number	Number of Components	%	Number	Number of Components	%
Insulin	31	31	96.88%	12	12	29.27%
Metformin	4	35	12.50%	16	28	39.02%
Other tablets	0	35	0.00%	12	40	29.27%

**Table 3:** Characteristics of the study group in terms of the occurrence of diabetic complications.

Diabetic Complications	Diabetes Type 1			Diabetes Type 2		
	Number	Number of Components	%	Number	Number of Components	%
No complications	19	19	59.38%	7	7	17.07%
Diabetic foot	4	23	12.50%	1	8	2.44%
Retinopathy	10	33	31.25%	5	13	12.20%
Nephropathy	12	45	37.50%	3	16	7.32%
Neuropathy	10	55	31.25%	4	20	9.76%
Other complications	20	75	62.50%	39	59	95.12%

**Table 4:** Characteristics of the study group in terms of the occurrence of chronic diseases.

The Occurrence of Chronic Diseases	Diabetes Type 1			Diabetes Type 2		
	Number	Number of Components	%	Number	Number of Components	%
NO	2	2	6.25%	2	2	4.88%
Heart diseases	4	6	12.50%	11	13	26.83%
Other circulatory system diseases	11	17	34.38%	37	50	90.24%
Hypertension	11	28	34.38%	36	86	87.80%
Blood vessel diseases	6	34	18.75%	6	92	14.63%
Lung diseases	1	35	3.12%	7	99	17.07%
Digestive system diseases	6	41	18.75%	10	109	24.39%
Liver diseases	0	41	0.00%	4	113	9.76%
Urinary system diseases	5	46	15.62%	29	142	70.73%
Gout	4	50	12.50%	8	150	19.51%
Thyroid diseases	6	56	18.75%	13	163	31.71%
Nervous system diseases	4	60	12.50%	2	165	4.88%
Systemic diseases	6	66	18.75%	23	188	56.10%
Osteoarticular	3	69	9.38%	19	207	46.34%
Joint degeneration	5	74	15.62%	10	217	24.39%
Blood and coagulation diseases	6	80	18.75%	13	230	31.71%
Eye diseases	7	87	21.88%	0	230	0.00%
Mood changes	2	89	6.25%	2	232	4.88%
Infectious diseases	1	90	3.12%	3	235	7.32%
Rheumatic diseases	0	90	0.00%	1	236	2.44%
Osteoporosis	5	95	15.62%	36	272	87.80%

**Table 5:** Spearman’s rank correlation between the concentrations of selected fibroblast growth factors (FGF-2, FGF-19, FGF-22, FGF-23), age, and BMI in: all participants (orange table); patients with type 1 and type 2 diabetes (blue table); patients with type 1 diabetes (green table); patients with type 2 diabetes (yellow table).

Variables	N	Rank Correlation Coefficient	*p*
**Age vs. FGF-2**	114	−0.06	NS
**Age vs. FGF-19**	114	−0.28	0.004
**Age vs. FGF-22**	114	−0.45	<0.001
**Age vs. FGF-23**	114	0.19	NS
**BMI vs. FGF-2**	114	−0.00	NS
**BMI vs. FGF-19**	114	−0.28	0.005
**BMI vs. FGF-22**	114	−0.46	<0.001
**BMI vs. FGF-23**	114	0.05	NS
**Variables**	**N**	**Rank Correlation Coefficient**	***p***
**Age vs. FGF-2**	73	−0.27	0.022
**Age vs. FGF-19**	73	0.22	NS
**Age vs. FGF-22**	73	−0.32	0.005
**Age vs. FGF-23**	73	0.08	NS
**BMI vs. FGF-2**	73	−0.04	NS
**BMI vs. FGF-19**	73	0.05	NS
**BMI vs. FGF-22**	73	−0.42	<0.001
**BMI vs. FGF-23**	73	0.06	NS
**Variables**	**N**	**Rank Correlation Coefficient**	***p***
**Age vs. FGF-2**	31	−0.26	NS
**Age vs. FGF-19**	31	0.24	NS
**Age vs. FGF-22**	31	−0.39	0.027
**Age vs. FGF-23**	31	0.21	NS
**BMI vs. FGF-2**	31	−0.14	NS
**BMI vs. FGF-19**	31	0.35	0.05
**BMI vs. FGF-22**	31	−0.29	NS
**BMI vs. FGF-23**	31	0.09	NS
**Variables**	**N**	**Rank Correlation Coefficient**	***P***
**Age vs. FGF-2**	41	−0.02	NS
**Age vs. FGF-19**	41	0.25	NS
**Age vs. FGF-22**	41	0.03	NS
**Age vs. FGF-23**	41	0.14	NS
**BMI vs. FGF-2**	41	0.16	NS
**BMI vs. FGF-19**	41	−0.35	0.022
**BMI vs. FGF-22**	41	−0.30	NS
**BMI vs. FGF-23**	41	−0.26	NS

**Table 6:** Spearman’s rank correlation between the concentrations of selected fibroblast growth factors (FGF-2, FGF-19, FGF-22, FGF-23) and HBA1C level and BMI in patients with diabetes type 1 and 2 diabetes.

Variables	N	Rank Correlation Coefficient	*p*
**HBA1C vs. FGF-2**	73	0.16	NS
**HBA1C vs. FGF-19**	73	−0.09	NS
**HBA1C vs. FGF-22**	73	−0.13	NS
**HBA1C vs. FGF-23**	73	0.64	<0.001

**Table 7:** Multivariate regression analysis for FGF-2.

	Independent Variable	β	R2	*p*	*p* for Model	F
FGF-2	diabetic foot	0.112965	0.263	NS	NS	0.850
	retinopathy	−0.110895		NS		
	nephropathy	−0.075255		NS		
	neuropathy	0.047224		NS		
	or other chronic diseases? Yes/No	0.070175		NS		
	heart diseases	−0.126949		NS		
	other circulatory system diseases	−0.138517		NS		
	arterial hypertension—YES/NO	0.220509		NS		
	blood vessel diseases	−0.079959		NS		
	lung diseases	−0.083051		NS		
	systemic diseases, digestive system	0.116100		NS		
	liver diseases	0.084055		NS		
	systemic diseases, urinary tract	0.586893		0.037		
	gout	−0.166920		NS		
	thyroid diseases	0.020166		NS		
	systemic diseases nervous	−0.012424		NS		
	systemic diseases osteoarticular	−0.170370		NS		
	joint degeneration	0.166763		NS		
	blood and systemic diseases, clotting	−0.004424		NS		
	eye diseases	0.020460		NS		

β—standardized coefficient in the regression equation, R2—coefficient of determination, *p*-value of the significance coefficient; FGF-2—fibroblast growth factor. NS—not significant.

**Table 8:** Multivariate regression analysis for FGF-23.

	Independent Variable	β	R2	*p*	*p* for Model	F
FGF-23	diabetic foot	0.148754	0.301	NS	NS	1.024
	retinopathy	−0.250782		NS		
	nephropathy	0.150875		NS		
	neuropathy	0.194741		NS		
	or other chronic diseases? Yes No	0.055626		NS		
	heart diseases	0.095206		NS		
	other circulatory system diseases	−0.055029		NS		
	arterial hypertension	0.108258		NS		
	blood vessel diseases	−0.034295		NS		
	lung diseases	0.136807		NS		
	systemic diseases, digestive system	0.032524		NS		
	liver diseases	0.012118		NS		
	systemic diseases, urinary tract	0.241291		NS		
	gout	−0.356860		0.024		
	thyroid diseases	0.213651		NS		
	systemic diseases nervous	−0.101706		NS		
	systemic diseases osteoarticular	0.031397		NS		
	joint degeneration	0.311688		NS		
	blood and systemic diseases, clotting	−0.034704		NS		
	eye diseases	−0.108087		NS		

β—standardized coefficient in the regression equation, R2—coefficient of determination, *p*-value of the significance coefficient; FGF-23—fibroblast growth factor.

**Table 9:** ANCOVA analysis for FGF-19 and FGF-23.

FGF-19	F	*p*
Age	0.842	NS
BMI	0.708	NS
Group	3.362	0.038
Systemic diseases, urinary tract	2.440	NS
Joint degeneration	0.034	NS
**FGF-23**	**F**	***p***
Age	0.118	NS
BMI	2.604	NS
Systemic diseases, urinary tract	72.3300	<0.001
Joint degeneration	550.575	<0.001

## Data Availability

The data used to support the findings of this study are not available because they are the property of the Pomeranian Medical University in Szczecin. Participants in the study did not consent to the disclosure of data outside the publication. All data generated or analyzed during this study are included in this article.

## Supplementary Materials

The following supporting information can be downloaded at: https://www.mdpi.com/article/10.3390/ijms26178754/s1.
