# Supplementary material for: The Promising Role of Selected Fibroblast Growth Factors as Potential Markers of Complications in Type 1 and Type 2 Diabetes
Source: Int J Mol Sci. 2025 Sep 8;26(17):8754. doi: 10.3390/ijms26178754 (PMC12429062; doi:10.3390/ijms26178754)
Supplement: Supplementary file 1 [file ijms-26-08754-s001.zip › ijms-3784213-supplementary.pdf]

Supplementary Table S1. Results of biochemical tests in patients with type 1 diabetes.

Diabetes type 1	Mean	SD	Median	IQR	Min	Max
Hb1ac concentration [ng/ml]	3378	15336	107	78,1	16,2	85220
Albumin concentration [g/dl]	4,64	1,27	4,44	2,16	2,76	7,5
Total protein concentration [g/l]	58,6	12,5	56,6	12,8	32,3	98,9
Urea concentration [mg/dl]	45,3	23,1	44,4	18,1	5,56	133
Uric acid concentration [mg/dl]	3,06	1,4	2,68	1,03	1,43	8,66
Creatinine concentration [mg/dl]	1,1	0,849	0,868	0,581	0,051	4,15
Direct HDL concentration [mg/dl]	34,8	13,6	35,2	14,4	5,81	58,1
Triglyceride concentration [mg/dl]	231	179	204	196	13	943
Glucose concentration [mg/dl]	174	71,2	172	91,8	58,2	389
Cholesterol concentration [mg/dl]	116	35,2	104	48,9	64,9	201

SD-standard deviation, IQR-interquartile range, Min-minimum value, Max - maximum value

Supplementary Table S2. Results of biochemical tests in patients with type *2* diabetes.

Diabetes type 2	Mean	SD	Median	IQR	Min	Max
Hb1ac concentration [ng/ml]	220	350	109	51,9	55,8	1727
Albumin concentration [g/dl]	6,49	1,54	6,98	2,44	3,43	8,83
Total protein concentration [g/l]	63,3	14,6	62,4	16,2	33,6	95,9
Urea concentration [mg/dl]	66,8	28,7	68,1	34,7	0	122
Uric acid concentration [mg/dl]	3,89	2,52	3,3	1,34	1,52	17,9
Creatinine concentration [mg/dl]	1,4	0,845	1,2	1,04	0,249	4,22
Direct HDL concentration [mg/dl]	36	14,1	35,5	18	1,06	64,5
Triglyceride concentration [mg/dl]	226	126	215	100	13	696
Glucose concentration [mg/dl]	147	44	141	53,2	77,2	263
Cholesterol concentration [mg/dl]	105	31,4	104	42,6	55,3	201

SD-standard deviation, IQR-interquartile range, Min-minimum value, Max - maximum value

Supplementary Table S3. FGF-2, FGF-19, FGF-22, and FGF-23 concentrations [pg/ml] consider group membership.

FGF-2						
Group	Mean	SD	Median	IQR	Min	Max
Control	153	119	101	157	39,1	508
Type 1 diabetes	130	65,7	117	47,9	58,7	349
Type 2 diabetes	104	47,2	98,6	42,6	51,8	257
**FGF-19**						
Group	Mean	SD	Median	IQR	Min	Max
Control	370	310	294	169	109	1671
Type 1 diabetes	188	259	143	54,4	47,8	1560
Type 2 diabetes	163	41,4	164	45562	68,1	322
**FGF-22**						
Group	Mean	SD	Median	IQR	Min	Max
Control	628	872	230	577	35125	4055
Type 1 diabetes	285	375	168	171	49,9	1925
Type 2 diabetes	260	422	98,5	75,5	0,564	1658
**FGF-23**						
Group	Mean	SD	Mediana	IQR	Min	Max
Control	558	622	307	385	23377	2908
Type 1 diabetes	554	608	310	209	205	2209
Type 2 diabetes	511	533	286	123	211	2301

SD-standard deviation, IQR-interquartile range, Min-minimum value, Max-maximum value.

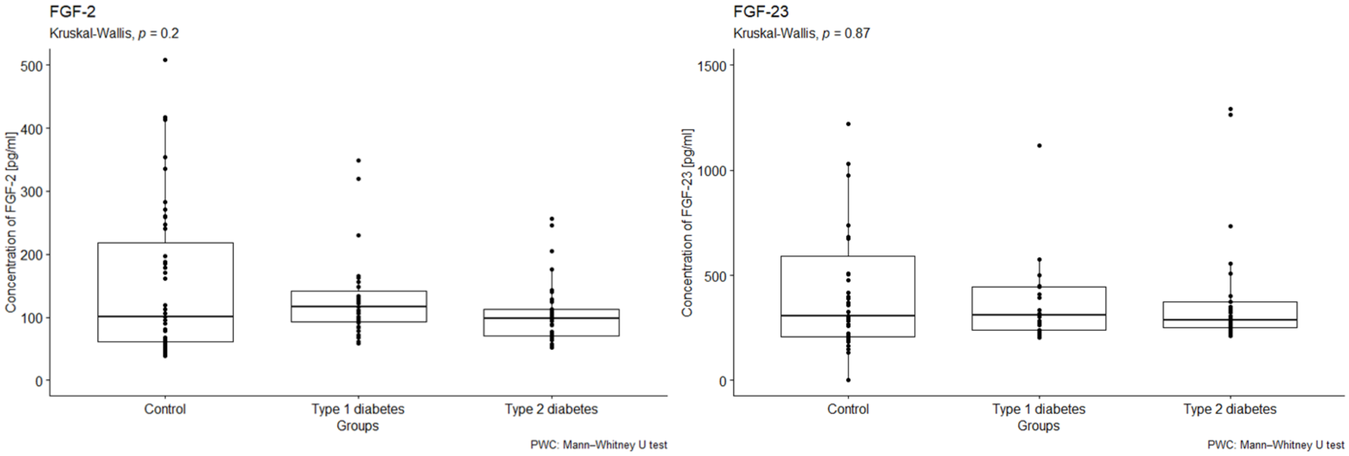

Supplementary Figure S1. Comparison of FGF-2 and FGF-23 concentration [pq/ml] in the EDTA of people with type 1 and 2 diabetes and the control group. Kruskal-Wallis rank ANOVA analysis of the relationship between FGF-2 concentration and group membership (p = 0.2) and (p = 0.87). The group composition included 41 individuals in the control group, 33 individuals with type 1 diabetes, and 40 individuals with type 2 diabetes.

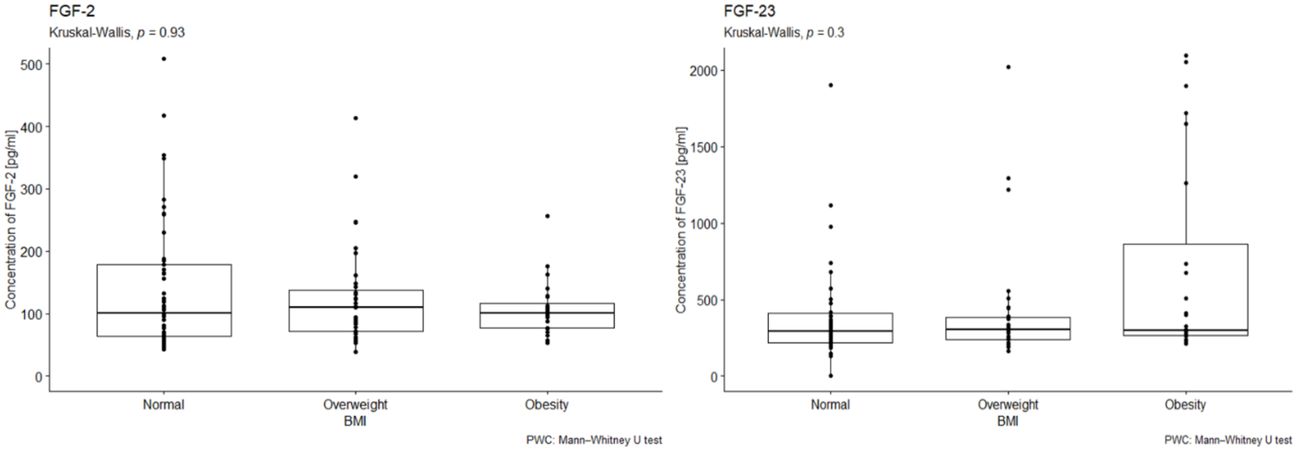

Supplementary Figure S2. Comparison of concentration according to body mass index. Kruskal-Wallis rank ANOVA analysis of the relationship between FGF-2 and FGF-23 concentration and BMI (p = 0.93) and p = 0.3.Normal- 53 person; Overweight-35 person; Obesity-28 person
